# Yeasts Inhabiting Extreme Environments and Their Biotechnological Applications

**DOI:** 10.3390/microorganisms10040794

**Published:** 2022-04-09

**Authors:** Claudia Segal-Kischinevzky, Lucero Romero-Aguilar, Luis D. Alcaraz, Geovani López-Ortiz, Blanca Martínez-Castillo, Nayeli Torres-Ramírez, Georgina Sandoval, James González

**Affiliations:** 1 Departamento de Biología Celular, Facultad de Ciencias, Universidad Nacional Autónoma de México, Avenida Universidad 3000, Coyoacán, Mexico City 04510, Mexico; claudiasegal@ciencias.unam.mx (C.S.-K.); lalcaraz@ciencias.unam.mx (L.D.A.); blancacastillo@ciencias.unam.mx (B.M.-C.); nayeli.torres@ciencias.unam.mx (N.T.-R.); 2 Departamento de Bioquímica, Facultad de Medicina, Universidad Nacional Autónoma de México, Avenida Universidad 3000, Coyoacán, Mexico City 04510, Mexico; lusromaguila@bq.unam.mx; 3 Subdivisión de Medicina Familiar, Facultad de Medicina, Universidad Nacional Autónoma de México, Avenida Universidad 3000, Coyoacán, Mexico City 04510, Mexico; geovani.lorz@fmposgrado.unam.mx; 4 Laboratorio de Innovación en Bioenergéticos y Bioprocesos Avanzados (LIBBA), Unidad de Biotecnología Industrial, Centro de Investigación y Asistencia en Tecnología y Diseño del Estado de Jalisco AC (CIATEJ), Av. Normalistas No. 800 Col. Colinas de la Normal, Guadalajara 44270, Mexico; gsandoval@ciatej.mx

**Keywords:** extreme habitats, extremophilic yeasts, stress response, yeast biotechnology, yeast identification

## Abstract

Yeasts are microscopic fungi inhabiting all Earth environments, including those inhospitable for most life forms, considered extreme environments. According to their habitats, yeasts could be extremotolerant or extremophiles. Some are polyextremophiles, depending on their growth capacity, tolerance, and survival in the face of their habitat’s physical and chemical constitution. The extreme yeasts are relevant for the industrial production of value-added compounds, such as biofuels, lipids, carotenoids, recombinant proteins, enzymes, among others. This review calls attention to the importance of yeasts inhabiting extreme environments, including metabolic and adaptive aspects to tolerate conditions of cold, heat, water availability, pH, salinity, osmolarity, UV radiation, and metal toxicity, which are relevant for biotechnological applications. We explore the habitats of extreme yeasts, highlighting key species, physiology, adaptations, and molecular identification. Finally, we summarize several findings related to the industrially-important extremophilic yeasts and describe current trends in biotechnological applications that will impact the bioeconomy.

## 1. Introduction

**Figure 1:**
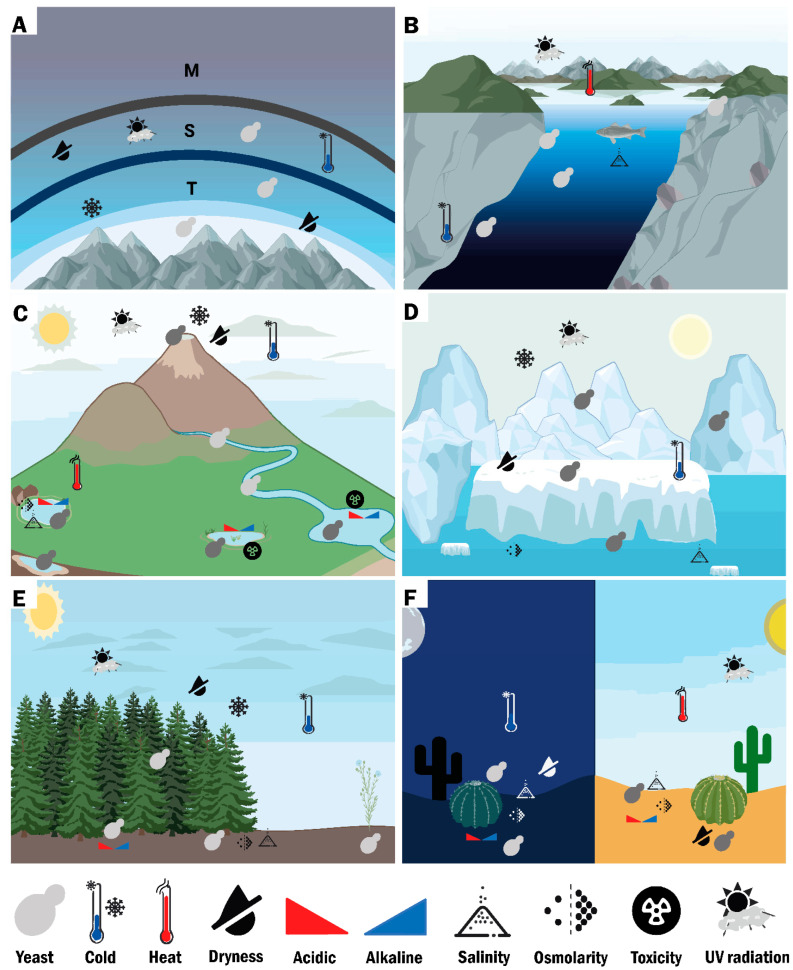
Representative scheme of yeasts in atmospheric, aquatic, and terrestrial environments. (**A**) In the atmospheric environment, yeasts have been found in the air of the highest mountains on Earth, the troposphere (T), even in the stratosphere (S), an environment of conditions of extreme cold, dryness, low atmospheric pressure, and high ultraviolet (UV) radiation. Yeasts are unlikely to proliferate in the air, viability is lost as height increases, but spores of some species can remain dormant and germinate later in favorable conditions. (**B**) In saltwater aquatic environments, yeasts can be found in the depths of the oceans, on the sea surface, in aquatic plants, in animals, etc. The conditions in this environment include combinations of temperature, atmospheric pressure, salinity or UV radiation. (**C**,**D**) In freshwater aquatic environments, yeasts have been found in rivers, lagoons, lakes, estuaries, glaciers, aquifers, geysers, etc. These environments may present combinations of conditions of cold, heat, dryness, acidic, alkaline, salinity, osmolarity, UV radiation, or toxicity (sites contaminated with industrial waste; e.g., heavy metals, chemicals, etc.). (**E**,**F**) In the terrestrial environment, yeasts have been isolated from soils, rocks, plants, animals, mountains, deserts, etc. The terrestrial environment presents combined conditions of cold, heat, dryness, acidic, alkaline, salinity, or UV radiation. Symbols for different extreme conditions are shown at the bottom of panels (**A**–**F**). Panel (**C**), based from Buzzini et al., 2018 [15]. Created using BioRender.com, accessed on 10 February 2022.

The evolutionary history of the first simple unicellular fungi in aquatic environments dates back more than ~1000 million years ago (Ma) [1,2]. However, fungi in terrestrial environments have been reported to have evolved over ~600 Ma, giving rise to the two largest fungal groups: Ascomycota and Basidiomycota, which include yeasts [2,3,4,5]. The term “yeast” refers to microscopic unicellular or dimorphic fungi with a main unicellular stage in the environment. Yeast-like forms can be found in Saccharomycotina and Taphrinomycotina (Ascomycota), and in Agaricomycotina, Pucciniomycotina, and Ustilaginomycotina (Basidiomycota) [6,7,8,9,10]. Yeasts reproduce asexually by budding or fission, producing single cells, and have sexual structures not enclosed in a fruiting body [11]. Yeasts are primarily free-living decomposers, which help accelerate several fundamental processes in ecosystems, such as decomposition of organic matter, generation of biomass as a source of nutrients for other organisms, mineralization of nutrients, and participate in nitrogen and sulfur cycles [6,8,12]. Yeasts maintain various types of ecological interactions with other organisms (plants, other fungi, animals, algae, etc.) in the form of mutualists, parasites, pathogens, among others. They can release spores which are dispersed by wind, water, and vectors (other organisms), over long distances, even between continents. Their physiological and metabolic capabilities allow them to adapt to a wide variety of biomes, including extreme environments with conditions of either cold, heat, dryness, acidic, alkaline, salinity, osmolarity, toxicity, UV radiation, or in combination (Figure 1 and Table S1) [8,13,14,15].

Yeasts inhabiting extreme environments can be found in stratospheric air, hot springs, cold and deep seas, polar lands, glaciers, deserts, acidic and alkaline habitats, dry rocks, etc. [6,12,16,17,18,19,20,21]. Extreme environments can be permanent or temporary and have been defined differently over time [12]. According to Buzzini and collaborators (2018) [15], extreme environments can be classified as anthropocentric, microbiological, and zymo-centric. The anthropocentric vision includes those places with conditions that a human cannot tolerate and that to date have not been colonized; however, these sites may harbor organisms adapted to these harsh conditions. From a microbiological point of view, microorganisms that thrive in extreme environments are considered and have been classified as psychrophiles (optimal growth temperature below 10 °C); thermophiles (optimum growth temperature above 45 °C); xerophiles (living in conditions with low water availability), acidophiles (optimum pH below 5); alkaliphilic (optimum pH above 8); halophiles (inhabiting hypersaline conditions), osmophiles (inhabiting conditions of high osmolarity), radiophiles (resisting high levels of radiation), metallophiles (tolerating high concentrations of heavy metals), and which can also thrive or tolerate different ranges of temperature, pH, pressure, desiccation, and salinity in the same niche [12,15]. The zymo-centric point of view relates to prokaryotic and eukaryotic microorganisms that exhibit adaptations to extreme environments, allowing them to colonize environments with various unusual conditions for life, such as polyextremophiles (thriving in areas that combine several extreme conditions) [15]. Currently, it is difficult to define whether a yeast is extremophile or extremotolerant at first. Some authors suggest that the biology and ecology of the organisms should be deeply studied, which will avoid confusing the common terms “extremophile” or “extremotolerant” [12], that sometimes refers to life in permanently extreme environments (obligate extremophile) or life that may have evolved in habitats subjected to different ranges of changes (facultative extremophile). Therefore, it should be kept in mind that the fact of isolating or detecting a microorganism in an extreme environment does not mean that it is extremophile or extremotolerant. In the case of yeasts, it can be considered extremophilic if (i) it has been repeatedly isolated from an extreme habitat, (ii) if it shows physiological capacities that allow it to overcome the environmental stress from which it was isolated, and (iii) if it has optimal growth in the environmental niche corresponding to the conditions found in the extreme habitat. In contrast, the extremotolerant yeasts are those that grow under extreme physical or chemical conditions without reaching their optimum growth [12,15,22].

The metabolic diversity of several yeasts has been studied, including those that inhabit extreme environments, revealing numerous possibilities for the development of biotechnological applications in the areas of environmental (bioremediation, degradation of pollutants), biocontrol (crop protection, agricultural safety, probiotics), research in biomedical sciences (drug discovery, metabolism, drug resistance, elucidation of disease mechanisms), basic research in biological sciences (cellular and molecular biology, comparative and functional genomics, engineering of metabolic pathways, systems biology), protein production (proteins for pharmaceutical use, enzymes, hormones, vaccines, toxins), biocatalysis (pharmaceuticals, chemical intermediates with chiral structure, biotransformers), food and ingredients (enzymes, flavorings, pigments, amino acids, organic acids), and renewable energies (production of biofuels, lipases) [22,23,24,25,26]. Therefore, the isolation and identification of extreme yeasts are opening a panorama to study the limits in which life is possible for eukaryotes, providing new research in biotechnology, where they represent one of the most relevant groups of microorganisms [25,27,28].

This review addresses the importance of investigating yeasts inhabiting extreme environments and their coping adaptations. The review discusses the identification of yeasts using widely applied molecular tools and variations of next generation sequencing and highlights relevant research in extremophilic yeasts with biotechnological applications.

## 2. Yeasts in Extreme Environments: Metabolic and Adaptive Aspects

Yeasts generally have adaptations to cope with oxidative stress through gene expression, encoding enzymes, such as superoxide dismutases (SOD), catalases (CAT), glutathione peroxidases (GPX), peroxiredoxins (PRX), and glutathione S-transferases (GST), which counteract the reactive oxygen species (ROS), allowing cellular homeostasis [29,30,31]. Likewise, it has been observed that the GABA-shunt pathway (gamma-aminobutyric acid pathway) formed by the enzymes glutamate decarboxylase (GAD), GABA aminotransferase (GAT), and succinate semialdehyde dehydrogenase (SSADH), may play a crucial role in protecting against cell damage by different types of stress by restricting the production of reactive oxygen species intermediates (ROI) through the production of nicotinamide adenine dinucleotide phosphate (NADPH) [32,33,34,35]. Yeast GABA shunt NADPH production plays a key role in stress resistance [36,37]. Currently, a new negative role of alanine was uncovered in heat stress tolerance; alanine accumulation represses the *GAD1*, *UGA1*, and *UGA2* genes (GABA shunt pathway), which decreases intracellular NADPH [38].

**Figure 2:**
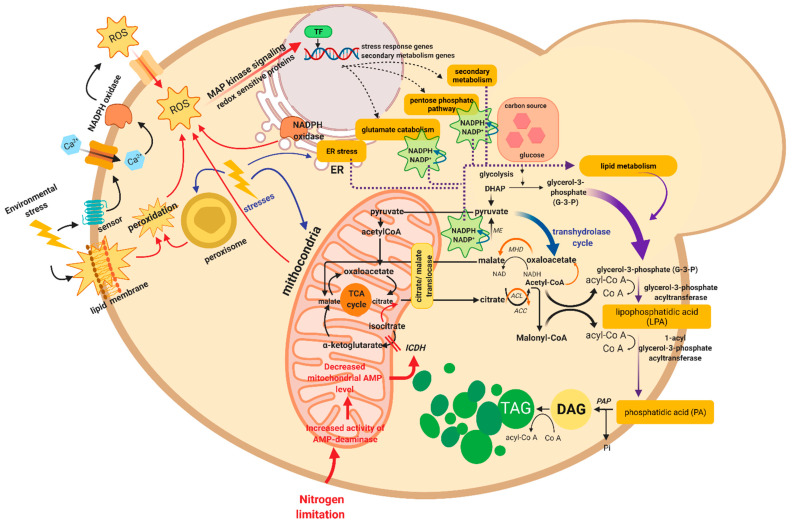
Representative scheme of the metabolic pathways activated under different stress conditions in non-*Saccharomyces* or oleaginous yeasts. The abbreviations correspond to reactive oxygen species (ROS), triacylglycerols (TAG), diacylglycerols (DAG), inorganic phosphate (Pi), nicotinamide-adenine dinucleotide phosphate (NADPH or NADP), NADPH oxidases (NOX), mitogen-activated protein kinases (MAPK, MAPKK, and MAPKKK), dihydroxyacetone phosphate (DHAP), transcription factors (TF), endoplasmic reticulum (ER), malic enzyme (ME), ATP-citrate lyase or synthase (ACL), acetyl-CoA carboxylase (ACC), malate dehydrogenase (MHD), and isocitrate dehydrogenase (ICDH). ROS accumulation generates oxidative stress, which increases secondary metabolites, the pentose phosphate pathway, glutamate catabolism, and ER stress. Low-temperature conditions increase neutral fatty acid synthesis from triacylglycerols (TAG), whereas at high temperatures, TAG desaturation increases. In oleaginous yeasts, oligotrophic conditions, such as nitrogen-limitation, induce lipogenesis and TAG accumulation in lipid drops (LD), alleviating lipotoxicity. Based from Patel, et al., 2016 and Shi et al., 2017 [43,44]. Adapted from “TAG synthesis”, by BioRender.com (2022). Retrieved from https://app.biorender.com/biorender-templates (accessed on 1 February 2022).

A relevant aspect is a relationship between the response to stress and lipid biosynthesis. Oleaginous yeasts produce high amounts of lipids (>20% of their biomass). It has been observed that several of them are considered extremophilic yeasts, for example, *Rhodotorula toruloides* (synonym of *Rhodosporidium toruloides*), *Debaryomyces hansenii*, *Kluyveromyces marxianus*, and others [39,40,41,42]. Interestingly, oleaginous yeasts can upregulate the lipogenic pathways under different types of biotic and abiotic stress (Figure 2) [43,44]. The stress response can be activated either by a negative environmental stimulus that produces ROS, through the action of nicotinamide adenine dinucleotide phosphate oxidases (NOX), or both, which utilize cytosolic NADPH as the electron donor to reduce extracellular O_2_ to O_2_^•−^, causing the subsequent formation of H_2_O_2_ [45,46]. Subsequently, MAP kinases pathways signaling (MAPK, MAPKK, and MAPKKK are sequentially activated by phosphorylation) reaching the nucleus, allowing activation of the expression of genes and enzymes to respond to the stress, including secondary metabolism, catabolism of pentose phosphate and glutamate, which together increase the production of NADPH and promote lipid biosynthesis by preventing damage by ROS [44,47,48]. It is known that lipids, and especially polyunsaturated fatty acids (PUFAs), may act as antioxidants or otherwise protective defense molecules in the stress response [49]. However, the direct mechanisms of ROS-mediated lipid accumulation are still unknown. Probably, the link between stress factors and lipid metabolism mediated by ROS is more evident in extremophilic yeasts. On the other hand, it has been observed that most yeasts can activate sporogenesis, particularly oleaginous yeasts can also accumulate triacylglycerols (TAG) under nitrogen-limited conditions in the presence of an abundant carbon source, such as glucose. Under this condition, the enzyme AMP-deaminase (AMPD) has been observed to break down adenosine monophosphate (AMP) into inosine monophosphate (IMP) and ammonium ions (NH_4_^+^), which allows them to obtain nitrogen and survive (Figure 2) [43,44,50,51]. However, the molecular mechanisms of non-conventional yeasts (non-*Saccharomyces* yeasts) under different types of stress in extreme environments are largely unknown. Most of the research that has attempted to decipher the molecular basis of the physiology of the extreme yeast is based on comparative studies with *Saccharomyces cerevisiae*, which in turn could exhibit different response adaptations to extreme conditions. The following highlights are some of the more remarkable adaptations discovered in yeasts that inhabit extreme environments.

### 2.1. Yeasts in Cold or Hot Environments

Psychrophilic and psychrotolerant yeasts have adaptations to inhabit regions of the Arctic, Antarctic, the high mountains of Asia, Europe, and America, low-temperature deserts, deep sea, among others. These environments have average temperatures below 5 °C, and most of them are associated with various stress factors such as low water and nutrient availability. However, yeasts living in cold environments possess physiological adaptations that decrease their growth rate and synthesize enzymes active at low temperatures and cryoprotective molecules [12,15,52,53]. An important characteristic of cold-adapted yeasts is the high synthesis of unsaturated fatty acids that ensures high plasma membrane fluidity, which is related to the degree of adaptability and survival of yeast in extremely cold environments or other stress conditions such as the yeast *Rhodotorula diobovatum* [54,55]. Another important characteristic of most cold-adapted yeasts is that they can proliferate at sub-zero temperatures as they manage to decompose organic compounds and accumulate high concentrations of tricarboxylic acid cycle metabolites, glycerol, and trehalose [56]. In the case of *R. frigidialcoholis* (synonym of *R.* JG1b), the adaptive response to cold temperatures in the Antarctic dry valley permafrost is through a variety of mechanisms including increased expression of the pentose phosphate pathway genes, increasing the production of exopolysaccharides, sphingolipids, unsaturated fatty acids, and carotenoids while coupled with a reduction in expression of growth, transcriptional and translational machinery genes [21].

On the other hand, thermophilic and thermotolerant yeasts have physiological strategies to adapt to the high temperatures of hot environments such as hot springs, deserts, hydrothermal vents, associations with warm-blooded animals, etc. Some of these habitats can reach temperatures above 45 °C, which compromises the stability of the cell membrane [12,57]. High-temperature environments can be accompanied by osmotic conditions, high sulfur or calcium carbonate levels, and acidic or alkaline conditions. Yeasts living in hot environments have adaptations that adjust the concentration of saturated fatty acids, thus maintaining an optimal degree of fluidity in the cell membrane. They also synthesize membrane-important phospholipids related to rapidly synthesizing and exchanging metabolites at high temperatures. They can also increase cytochrome concentrations, which correlates with enhanced mitochondrial respiration activity [8,12,15,58,59]. *Takashimella tepidaria* and *Ogataea thermophila* (synonym of *Candida thermophila*) are examples of thermophilic yeasts that grow from 47 to 51 °C [59,60].

Although yeasts can live in extremely cold or hot environments, most of them are mesophilic (optimal growth temperature of 20–25 °C), tolerating different temperature ranges and having physiological strategies that preserve their cellular integrity and functionality, allowing them to conquer diverse ecological niches on Earth [15,61]. In addition, psychrophilic or thermophilic yeasts may experience changes of temperature over the course of a day/night cycle (Figure 1F), suggesting the conservation of clock-gene homologues as were found in *Aureobasidium pullulans* [62]. However, little is known about temperature, circadian cycle, and the metabolism of psychrophilic and thermophilic yeasts.

### 2.2. Yeasts in Dry Environments

Xerophilic yeasts (sometimes called osmophilic) inhabit heat or polar deserts, sandy soils, mountain peaks, caves, or places with a high concentration of solutes, resulting in low water availability, which is expressed as water activity (a_w_ = the available aqueous activity expressed in mole fraction; pure water has a_w_ = 1, while any other solution has a_w_ < 1) [63]. Dry environments have low a_w_ because water is usually frozen, saturated with salts, or of limited availability in these locations. Yeasts isolated from these environments can tolerate water stress and release drought-resistant structures such as ascospores, teliospores, and chlamydospores that germinate in favorable conditions [15,64,65]. In vegetative form, they can synthesize a polysaccharide capsule that prevents desiccation [12,15,66]. Some yeasts that inhabit dry environments are *Aureobasidium namibiae*, *Candida thaimueangensis*, *Cladosporium sphaerospermum*, *Hanseniaspora opuntiae*, *H. uvarum*, *Sporobolomyces johnsonii*, *Starmerella apicola*, *Wallemia muriae*, *W. ichthyophaga* (synonym of *Candida apicola*), several of them isolated from desert regions [12,56,67,68,69]. In particular, *W. ichthyophaga* can grow with low a_w_, ranging from 0.95 to 0.77 [67,68].

Xerotolerant or osmotolerant yeasts can survive in environments of high osmotic pressures generated by the high concentration of organic solutes, particularly the sugars present in the nectar of flowers, honey, fruits, etc. Under hyperosmotic conditions (low a_w_) osmophilic yeasts increase their intracellular solute concentration by pumping inorganic ions found in the external environment (e.g., cadmium, iron, copper, and zinc) or by synthesizing compatible solutes (e.g., polyols), disaccharides, oligosaccharides, amino acids, quaternary amines, and betaines [15,69,70,71,72]. It has been reported that, in general, yeasts accumulate and utilize glycerol, trehalose, proline, arginine, or GABA as compatible osmolytes, which contribute to osmo-adaptation [35,70,73,74]. The genera *Pichia*, *Saccharomyces*, and *Zygosaccharomyces* are examples of osmotolerant yeasts and have been identified in foods with high concentrations of sugars (e.g., fresh fruits, nuts, commercial foods, etc.) [75]. In particular, *P. kudriavzevii* (synonym of *C. glycerinogenes*) accumulates large amounts of glycerol that serves as a compatible osmolyte to maintain water balance, restore cell turgor, and survive in hyperosmotic conditions [70]. In recent years, GABA accumulation has been shown to play an important role in resistance to osmotic stress in *P. kudriavzevii* [35].

### 2.3. Yeasts in Acidic and Alkaline Environments 

Acidophilic and acid-tolerant yeasts can live in acidic boreal and tropical soils, volcanic and some hot springs, gastric fluids, acidic rivers, and lakes. These locations reach a pH ranging from 1 to 3; however, acidophilic yeasts maintain an optimal cellular pH of 4.5–5.5, which allows them to carry out their biological processes. Acidophilic yeasts have physiological adaptations to decrease cell membrane density and have a significant gradient across the membrane that minimizes the movement of protons into the cell [12,15]. Likewise, yeasts inhabiting alkaline environments that present a pH between 8 and 10 (soils with soda, excrement, swamps, hot springs, hypersaline lakes, among others) manage to protect the macromolecules that compose their cellular organelles by regulating the cytoplasmic pH towards neutrality through a chemical gradient of protons across the membrane, avoiding any drastic changes in the cytoplasmic pH regardless of the external pH [66]. Yeasts such as *Cyniclomyces guttulatus* can inhabit the mammalian gastrointestinal tract, specifically the mucosa and the gastric pylorus, which have an acidic pH between 2 and 2.5. One strategy that allows them to remain in the gastrointestinal tract for long periods is the formation of ascospores [76]. Likewise, yeasts such as *Wickerhamomyces anomalus* can be found in acidic and alkaline environments, as they tolerate a wide range of pH between 2 and 12 [61,77].

### 2.4. Yeasts in Saline Environments

Halophilic and halotolerant yeasts inhabit places with 0.3 and 3.4 mol/L (2–20%) of sodium chloride (NaCl) such as seawater, salt lakes, solar salt flats, the Dead Sea and the Great Salt Lake, etc. [12,15,66,78]. In these saline environments, toxic concentrations of Na^+^ ions are higher than those of K^+^ ions, thus the mechanisms and transports that maintain the high intracellular K^+^/Na^+^ ratio are essential for the homeostasis of any halophilic yeast. Osmotic stress causes intracellular glycerol accumulation and decreases cell membrane permeability, leading to a decrease in biomass, which is reflected in an increase in energy expenditure required to keep internal osmotic pressure balanced [13,79]. *Hortaea werneckii* (black yeast-like fungi) is the eukaryotic model that best tolerates halophilic aquatic environments that exceed 20% salinity or conditions with more than 5 M NaCl. *H. werneckii* can produce melanin, fatty acids methyl ester compounds, diazirine, and azetidinone that allow it to compete for different extreme niches such as saline ones [68,80,81,82,83,84,85,86]. The physiological, biochemical, and molecular mechanisms enabling salt stress tolerance have been studied mainly in *Debaryomyces hansenii* and *Saccharomyces cerevisiae*. Both yeasts activate the high-osmolarity glycerol pathway (HOG), which increases glycerol production through the enzyme glycerol-3-phosphate dehydrogenase 1 (Gpd1) and the activity of SOD, CAT, GPX, PRX, and GST enzymes, which decrease oxidative stress under hypersaline conditions [70,87,88,89,90]. Activation of the HOG pathway (Sho1, Pbs2, Hog1, Ste11, Ssk1, Ssk2, Ypd1) is related to a loss of turgor leading to transient phosphorylation of the mitogen-activated protein kinase Hog1 (MAPK) [46,70,79,88,89,91,92,93]. Obligate halophilic yeasts, such as *Wallemia ichthyophaga*, have been observed to conserve components of the HOG pathway (WiPbs2, WiHog1A/B, WiSte11, WiSsk1, WiSsk2, WiYpd1) and overexpress hydrophobins that maintain cell wall thickness in saline environments [94,95].

### 2.5. Yeasts in Environments with Ultraviolet Radiation

Some yeasts can inhabit environments exposed to ultraviolet type B radiation (UVB, 280–320 nm) found at different atmospheric altitudes, polar regions, deserts, high mountains such as Los Andes, aquatic environments lacking natural shade, etc. Prolonged intense UV radiation exposure causes damage to organic molecules (nucleic acids, proteins, and lipids) and leads to the accumulation of ROS, reducing the growth rate of any microorganism [12,15,16,40,56]. However, radiophilic or radiotolerant yeasts that survive in environments with high UV radiation, in general, synthesize photoprotective compounds such as pigments, mycosporins, and antioxidants. The yeasts *Naganishia friedmannii* and *Exophiala* spp., are a model to study resistance to UV radiation in conditions simulating the stratosphere (temperature of −56.5 °C, pressure 5800 Pa, high exposure to UVB radiation, and osmotic condition). Both species show significant survival compared to other species, which is of great importance in astrobiology research [16,40]. Another interesting species is *Rhodotorula toruloides*, which resists even UVC radiation (100–280 nm); this strain was isolated from a volcanic area in the Atacama Desert with conditions of a high incidence of UV radiation, few sources of organic carbon, significant daily temperature variations, and osmotic conditions [40]. On the other hand, photoprotective compounds and radioresistance are present in non-pigmented yeasts. Phytoene and phytofluene (colorless carotenoids) have antioxidant capabilities with important biological effects over a wide range of conditions that allow cellular homeostasis [96].

### 2.6. Yeasts in Environments Contaminated with Heavy Metals

Metallophilic yeasts survive in toxic or contaminated environments with high concentrations of heavy metals (mercury, cadmium, arsenic, tin, cobalt, chromium, lead, nickel, zinc, or copper). These metals can accumulate in eroded tropical soils, industrial or mining wastewater, polluted rivers, etc. Heavy metals are toxic when their concentration exceeds a certain threshold. Metal toxicity affects the homeostatic pathway and causes oxidative stress [97,98], which alters enzyme and protein function and lipid peroxidation and leads to DNA damage [92]. In general, metallophilic yeasts tolerate high concentrations of heavy metals (0.1–200 mM) and are oligotrophic and synthesize extracellular redox enzymes that reduce metal ions [12,15]. Some yeasts, such as *Yarrowia lipolytica*, exhibit high tolerance to zinc and chromium through the formation of biofilms that counteract the effects of these heavy metals [12]. It has been suggested that biofilm formation is an innate means for yeasts to survive metal toxicity in the environment [99]. Biosorption or bioaccumulation of heavy metals by exopolysaccharides (EPS) is one of the important mechanisms contributing to heavy metal resistance traits in microorganisms. The EPS is crucial to the formation of biofilm and cell aggregates, which contribute to protecting cells from hostile environments [100]. It has been observed that *Zygosaccharomyces rouxii* accumulate heavy metals both inside the cell and on the cell surface through the expression of transporters, reductases, oxidases, and permeases [15,101].

### 2.7. Yeasts in Environments with Various Extreme Conditions

In general, yeasts that tolerate high incidence of UV radiation can often inhabit other stressful environments, which is why they are considered polyextremotolerant or polyextremophilic. It is common to find them in places with several extreme conditions: low temperature, scarce water availability, periodic freezing and thawing cycles, high osmotic, oxidative stress, among others. Polyextremophilic organisms have been suggested to be those that can tolerate and grow (not necessarily optimally) under multiple types of stress in places of extreme conditions [102]. Carotenoid-producing yeasts can be considered polyextremophiles, several of them are characterized by tolerating environmental factors with high doses of UV radiation, the presence of alcohols (ethanol, methanol, isopropanol, ethylene glycol, nicotine, and diphenylamine), and several stress factors [103,104]. However, uncolored yeasts are also polyextremophile, e.g., *Naganishia vishniacii* is resistant to high doses of UV radiation, low temperature, low pH, and scarce water availability [105]. It has been observed that one of the most important adaptations of polyextremophilic yeasts to counteract environmental effects is the presence of genes and enzymes involved in the production of trehalose (antioxidant), mycosporines, and carotenoids [57]; the latter blocks out certain wavelengths of light that would otherwise be damaging to the cell.

The black yeast-like fungi (dematiaceous fungi) or the so-called “black yeasts” (belong to Dothideomycetes, Pezizomycotina, Ascomycota) stand out as polyextremophiles, e.g., *Aureobasidium pullulans*, *Cryomyces antarcticus*, *Exophiala alcalophila*, *Hortaea werneckii*, *Phaeotheca triangularis*, *Trimmatostroma salinum*, and *Wallemia ichthyophaga* [15,81,106]. Black yeasts have developed a set of structural and functional adaptations that allow them to synthesize photoprotective compounds, antifreeze proteins, and lipids that generate changes in membrane fluidity [19,57,102,107,108,109]. Some species, such as *H. werneckii*, can withstand high concentrations of salt and tolerate high UV exposure, which was isolated from decomposed leaves on the Red Sea coast of Saudi Arabia [86,107]. *A. pullulans* is distributed in all climate zones [108], it is abundant in the phyllosphere, withstands hypersaline and glacial environments, among many other unusual conditions, such as contaminated water with heavy metals, foods preserved in salt, aviation fuel tanks, synthetic polymers, and PVC plastics [110,111,112,113,114,115,116,117,118,119,120]. *A. pullulans* is also an opportunistic human pathogen [121,122]. Another interesting example is *C. antarcticus* that resists exposure to temperatures close to 90 °C for one hour [123], osmotic stress, and radiation doses close to 1000 Gy. Under the aforementioned conditions, *C. antarcticus* can maintain high metabolic activity and does not suffer DNA damage. Due to these characteristics, *C. antarcticus* has been considered for astrobiological research, particularly for the biological exploration of Mars and the lithopanspermia theory [109,124], which proposes that the rocks that bombarded the Earth more than 4 billion years ago contained the organic molecules needed to form the building blocks of life.

Black yeasts are also found on exposed rocks, which represent a polyextreme environment to any form of life; these niches have conditions of oligotrophy, cold, heat, dry, and UV radiation (endolithic environment). These fungi involved in endolithic environments were recently grouped as rock-inhabiting fungi [20]. Melanin synthesis protects them from UV radiation, oxidative stress, drastic changes in temperature, and dehydration. Some black yeasts produce exopolysaccharides, which facilitate water retention and provide mechanical stability to the microbial community [19]. Species of the genus *Taphrina* have a dimorphic lifestyle; in its teleomorphic filamentous form, it proliferates exclusively as a biotrophic plant pathogen, while in anamorphic stages it can grow as a saprobe. Coleine and collaborators (2020) [19] suggested that this species may have adapted to life on rocks by permanently switching to an asexual saprotrophic lifestyle. This ability may be advantageous for survival and allow this species to explore new extreme ecological niches, such as rock-dwelling microbial communities [19]. Species like *Taphrina antarctica* exhibit adaptive strategies to overcome the negative effect of low temperatures (4–10 °C), namely, increased membrane fluidity, production of cold-shock and anti-freeze proteins, and cold-active enzymes [28,125].

## 3. Yeast Isolation and Molecular Tools

Less than 1% of the yeast species in nature have been discovered [6,126]. Therefore, the isolation of new wild yeasts is important, particularly those inhabiting extreme environments, as they are a biotechnological treasure [127]. Before collecting, it is important to review the Nagoya protocol on access to genetic resources and biological diversity, which aims at sharing the benefits arising from the utilization of genetic resources in a fair and equitable way (https://www.cbd.int/abs/about/ (accessed on 10 February 2022)). For intellectual property, and adherence to inter-institutional standards and international biodiversity treaties, the collection of yeasts should be properly documented and labeled with photographs, location, date, temperature, altitude, depth, site coordinates, the season of the year, type of climate, environment, etc. [128,129,130]. Likewise, biosafety measures must be taken, especially when collecting samples from cold environments that could harbor pathogens, such as viruses, bacteria, and parasites, that were buried for thousands of years and are unknown or dangerous to humans [131]. The isolation of specific extreme yeasts from a given natural habitat requires different growth conditions (Table S2). However, collecting and isolation is only the first step in the research, as later it will be necessary to focus on the identification of each yeast isolate.

The identification of extreme yeasts is of great interest to industry due to their diverse biotechnological applications [25,132,133]. Currently, more than 2000 yeast species have been isolated, identified, and classified according to MycoBank and some authors [9,129,134]. Many of these yeasts have been isolated from extreme environments (Table S1). For molecular identification, there are sequencing-free techniques, such as polymerase chain reaction (PCR) using primers and hybridization probes, random amplified polymorphic DNA (RAPD), amplified fragment length polymorphisms (AFLPs), restriction fragment length polymorphisms (RFLPs), DNA-fingerprinting, Real-Time PCR, MALDI-ToF mass spectrometry, and others [135,136,137,138,139,140,141]. However, the current gold standard for rapid identification and phylogenetic assignment of yeast isolates relies on PCR amplification of targeted sequences, such as the 18S rRNA gene and the internal transcribed spacer (ITS) located between the small subunit (SSU) and large subunit (LSU) of ribosomal RNA genes [129]. Historically, yeasts were directly identified by sequence analysis of D1/D2 domains of the LSU then homolog matching sequences to databases (e.g., GenBank), and phylogenetic placement [142,143,144,145]. Therefore, the development of databases (barcode) from D1/D2 and ITS permits many laboratories to reliably identify yeasts [135,146,147,148]. Included in those ITS and LSU sequence databases are yeasts from extreme environments (e.g., psychrophilic, xerophilic, alkalitolerant, thermotolerant, halophilic) [86,145,149,150,151,152,153].

Whole genome shotgun (WGS) sequencing is a fast-growing alternative for describing the taxonomy, coding genes, and metabolic pathways. The dropping cost of sequencing and the constant development of next-generation sequencing (NGS) technologies enable large-scale comparative genomics [154]. The NGS enabled yeasts and fungi phylogenomics [155], even the phylogenetic placement of previously unknown fungi phyla from single-cell genomics [156], and exploration of phenotypic diversity among populations [157]. Currently (March 2022), there are 3326 fungal genomes available at the NCBI, most of them within the Ascomycota (2345) and Basidiomycota (737). A fantastic resource for exploring fungal genomes is the MycoCosm portal maintained by the Joint Genome Institute [158]. One easing factor for sequencing yeast genomes is their relatively small average genome size ranges with Ascomycota (36.91 Mb) and Basidiomycota (46.48 Mb) [159]. Small genome sizes enable the sequencing of multiple isolates in the same run (multiplexing), saving time and resources [160,161]. However, there are bottlenecks in the WGS, mainly in the analysis, with researchers mocking the situation of the US Dollars 1000 genome and the USD 100,000 analysis [162]. The analysis “costs” include adequate computing facilities, trained personnel, and the unvaluable time invested (i.e., learning curves). A recent review summarizes the fine details of genome analysis from quality control, assembly (i.e., de novo, reference guided, hybrid assemblies), gene calling, annotation, ploidy assessment, phylogenetic placement, and comparative genomics [163]. Nevertheless, this WGS approach offers a valuable opportunity for the identification of yeasts from extreme environments [163,164].

Remarkably, metagenomics has allowed the exploration of microbial diversity. The first step in metagenomics involves metagenomic DNA/RNA extraction, so it frees up the need for cultivation, however DNA extraction needs to be standardized to compare between different studies. Metagenomic DNA could be used as input for shotgun metagenomics (SMG) or as a template for targeted amplified loci (e.g., 16S, 18S rRNA genes, ITS). SMG unlocks the full microbial community (virus, bacteria, archaea, and eukaryotes) along with their coding genes, without PCR amplification bias and primer design. If starting from metagenomic RNA it is called metatranscriptomics, it is also possible to do either SMG or amplicon-based metatranscriptomics. Direct RNA sequencing reflects the metabolically active community members (rRNA) and their expressed genes at the sampled time (mRNA), having the drawback of RNA lability [165,166,167,168]. In low diversity environments it is possible to use SMG to assemble genomes, metagenome assembled genomes (MAG), and even detect hybridization of species in yeast genomes [169]. However, the sequencing coverage does not allow recovering of whole fungal genomes in high diversity environments where they are in small abundance (from 0.2 to ~1%), such as in the Atacama Desert halite nodules and evaporitic rocks [170,171]. Even in non-extreme environments, such as soil and plant roots, there are reports of as few as 3.83% SMG assigned to fungal sequences [172]. Some technologies enable high quality sequenced genomes, such as hybrid sequencing and assembly of long and short sequencing reads [173,174,175], and high-throughput chromosome capture (Hi-C) in metagenomic samples [176,177]. However, a current limitation of SMG for biodiversity purposes, is the need for reference genomes, to assign species in a phylogenomic way (i.e., using core genome alignments and comparisons), thus highlighting the relevance of culturomics or massive cultivation, sequencing, and identification of isolates.

Targeted loci amplified from metagenomic DNA/RNA are the choice for describing large scale fungal diversity, though limited to answering meta-taxonomic identification. The ITS is the largest repository for fungal microbiome analysis and is used to perform taxa assignments, there are comprehensive databases such as UNITE with >1 × 10^9^ sequences representing 120,183 fungal species hypothesis (SH; 98.5% sequence identity clusters, UNITE version 8.3) [153]. Comparing the 2345 Ascomycota genome sequences against the 5515 SH representative clusters classified as Ascomycota illustrates the exponential growth of amplicon-based metagenomics and the gap of knowledge in reference genomes, highlighting the relevance of cultivation strategies for less studied yeasts. LSU and SSU rRNA genes do not provide robust phylogenetic assignments below phylum, class, and order levels to the fungi, thus the rapid growth of ITS capable of genus and species resolution, but conciliation between phylogenetic placement of databases such as UNITE and SILVA is relevant and it is on-going (LSU and SSU database) [153,178]. In the last two decades, yeast identification has been modified or complemented as technology advances. However, gene and genome sequence analyses are redefining many genera and species, including yeasts inhabiting extreme environments, some of which were initially misclassified.

The biological, ecological, and evolutionary relevance of using metagenomic approaches studying yeasts in extreme environments is to shed light on their roles as community members, describe their ecological interactions, metabolic contribution, and evolutionary relationships. There are successful examples of amplicon-based or SMG in yeasts/fungi research in food fermentation such as kombucha [179], pulque [180,181], cheeses [182], testing the role of yeasts as lichens symbionts [183], in saline lakes [184], acidic soils [185], mine wastelands [186], plastic fabric degradation [187], and desertic saline environments [170,171].

## 4. Biotechnological Applications of Extremophilic Yeasts

Non-*Saccharomyces* yeasts represent a poorly-explored field with great potential for biotechnology use in the production of value-added compounds such as biofuels (bioethanol), carotenoids, flavor enhancers, polyalcohols (xylitol), recombinant proteins, enzymes (pectinases, proteases, amylases, lipases, xylanases, laccases, esterases, etc.), hormones, vaccines, and toxins that can be used for biological pest control [22,23,24,25,26].

Among the extremophilic yeasts of biotechnological interest is *Zygosaccharomyces rouxii*, an aromatic yeast isolated from chili sauce that can grow in concentrations of 60–70% glucose and produces 2-phenylethanol or rose honey aroma [188,189]. From the same genus is *Z. bailii* isolated from vinegar, tea, and wine fermentation processes. This yeast tolerates high acetic acid concentrations and relatively high temperatures [190]. In addition, *Z. bailii* produces the alcohols farnesol (natural pesticide against mites), geraniol, nonanol, and nerolidol-2, some esters, organic acids, and aldehydes using sorghum as substrate [191]. Some *Kluyveromyces* species also stand out, such as *K. marxianus*, which is characterized by its rare ability to ferment lactose, with ethanol being the final product. The yeasts *K. marxianus* and *K. lactis* are thermotolerant (45–52 °C), which also ferment lactose and produce ethanol [192,193]. Likewise, the psychrophilic yeast *Glaciozyma antarctica* PI12 stands out for producing cold-active enzymes that have activity at low temperatures, which is of great importance in the food industry [194,195,196].

Another promising genus for biotechnology is *Rhodotorula*; one of its most relevant species is *R. glutinis*, which was isolated from sour milk. It is a red oleaginous yeast that produces carotenoids (vitamin A precursors, antioxidants, used in the food industry as colorants for beverages, food, salmon, and in the cosmetic industry) [197,198]. *R. glutinis* can proliferate at low temperatures (5 °C) and in the presence of 10% NaCl (1.7 M). It uses a wide variety of carbon sources, such as glucose, galactose, sucrose, maltose, trehalose, ethanol, glycerol, hexadecane, cellulose, and hemicellulose, the latter two coming from lignocellulose. It can also proliferate in wastewater from starch production and distilleries. Currently, the global carotenoid market is developing and is expected to reach USD 1–2 billion in 2022–2026 [199,200].

On the other hand, lipids are value-added products that, in addition to being raw materials for biofuels, have various applications in the cosmetic, pharmaceutical, and food industries. Some yeasts are capable of producing lipids essential for health care, such as omega-3 (eicosapentaenoic), omega-6 (docosahexaenoic), and lipids of interest to the industry (linoleic acid and ricinoleic acid), therefore microbial lipid production has increased [201]. Some oleaginous yeasts can metabolize various carbon sources, including organic residues, such as sugarcane bagasse, corn stover, starch wastewater, and olive mill wastewater, and accumulate up to 70% of their dry weight in the form of lipids. Although bioethanol is currently produced from plants, the world production of biofuels is still insufficient. Therefore, metabolic engineering of oleaginous yeasts represents an opportunity to increase the feedstock (lipids) and compensate supply for the global energy demand [202,203]. Lipids derived from animals are also not intended to supply the increasing needs for biologically important lipids, such as omega-3 and omega-6 that rely on marine oily fish that feed on marine phytoplankton [204], which are diminishing due to increasing global temperature, leading to reduced contents of omegas in caught fish. Unfortunately, the supplies of omegas from our oceans are falling because of overfishing [205]. To this end, various yeasts have been proposed to enhance lipids production. For example, *R. mucilaginosa* 50-3-19/20B is a promising extreme yeast in the production of bio-oils and biosurfactants that was collected from the Mid-Atlantic Ridge (deep-sea sediments) [18]. *R. toruloides* is a yeast with the ability to produce lipids and carotenoids, both from acetyl-CoA. This yeast has been isolated from pinewood pulp, seawater, and acidic wastewater (pH 2.5–3) [203,206]. It metabolizes hexoses and pentoses, such as xylose derived from the depolymerization of cellulose and hemicellulose. This yeast can also assimilate p-coumaric acid (4-hydroxy-cinnamic acid), which derives from the cell wall of grasses. Its importance lies in its ability to produce bisabolene, the immediate precursor of diesel D2, and amorphadiene, the precursor to the antimalarial drug artemisinin [207]. The demand for lipids that support human health has grown, and yeasts represent an excellent source of supply, thus the interest in obtaining them has increased, and currently, the global market for the production of omega-3 and omega-6 of microbial origin is worth USD 13 and USD 2 billion, respectively [208].

Table 1 shows several examples of yeasts that inhabit extreme environments with biotechnological applications.

## 5. Conclusions and Perspectives

Extreme yeasts conserve general pathways that respond to cell-damaging oxidative stress. They have particular strategies to proliferate in conditions in which non-extremophilic yeasts do not thrive. Among the most prominent adaptations are: (i) the synthesis of exopolysaccharides, sphingolipids, and saturated and unsaturated fatty acids; (ii) the biosynthesis of antifreeze proteins; (iii) the structuring of the cell wall and membrane; (iv) the production of compatible solutes; (v) efficient proton transport; (vi) synthesis of pigments; (vii) formation of biofilms, among others. However, the metabolic pathways that mitigate oxidative stress need to be studied more in depth to uncover the molecular mechanisms. Currently, the interest in the study of adaptive responses related to environmental stress in yeasts has increased significantly. The comparative genomics studies allow the identification of orthologous genes between yeasts for further study. Unfortunately, genomic information is still unavailable for many extreme yeasts. Therefore, further investigations should include genome sequencing to enable the identification of resistance-related genes. In the future, deeper molecular research will be possible, including gene deletion, overexpression, and heterologous gene expression, allowing a better understanding of the metabolic pathways and molecular mechanisms to cope with multiple stress conditions.

The rapid development of metagenomics and cultivation-free methods to describe environmental yeasts genes and diversity is an incentive to keep up the large-scale isolation of strains. Metagenomics is enriched by sequencing complete genomes from diverse environments and phylogenetic origins. Because fine molecular, ecological, and evolutionary analyses depend on the reference genomes and other sequence databases, thus classic microbiology along molecular descriptors is complementary. De novo sequencing of extreme yeasts will expand the understanding of the molecular basis of their biodiversity, adaptations to their niches, phylogenomics, gene novelty, and metabolic diversity, enabling the study of the molecular basis of yeast physiology in extreme environments. Targeted amplicon sequencing is the choice for biodiversity and species description research (ITS, rRNA). Shotgun metagenomics is the choice for studying yeasts and microbial community diversity and adaptations to extreme environments. Finally, we think that nowadays, yeast multidisciplinary approaches should include microbial ecology, microbiology, physiology, and molecular biology (e.g., metagenomics, metatranscriptomics, metaproteomics, and metabolomics). Then, the partnership between disciplines would accelerate the discovery of new strategies and adaptations conserved in yeasts inhabiting extreme environments, which will revolutionize microbial biotechnology.

Several extreme genera of yeasts that stand out in the literature with biotechnological potential are: *Aureobasidium*, *Candida*, *Cryomyces*, *Cryptococcus*, *Debaryomyces*, *Exophiala*, *Hortaea*, *Metschnikowia*, *Naganishia*, *Rhodotorula*, *Wallemia*, *Wickerhamomyces*, *Yarrowia*, and *Zygosaccharomyces*. Academia and industry have great interest in studying the cellular strategies of these genera due to their distinctive capacity to grow and metabolize under extreme conditions. However, most of the current research focused on the study of extreme yeasts is carried out under controlled laboratory conditions that do not resemble natural habitats or extreme environments, underestimating the adaptability of each yeast. Their natural conditions could encompass multiple biotic and abiotic factors that change during different seasons of the year in each ecological niche. Therefore, much remains to be learned about the physiological adaptations conserved by extreme yeasts, taking into account the natural conditions of each niche, which could give a better understanding and applicability in the biotechnology industry.

## Figures and Tables

**Table 1:** Biotechnological applications of extremophilic yeasts.

Extremophilic Yeasts	Conditions	Products	References
*Aureobasidium pullulans*	Glucose-seawater, 30 °C	Siderophores (0.7–1.1 mg mL^−1^) Amylase (58.5 U mg mL^−1^)	[209]
*Candida antarctica*	Heterologous expression of CalB and LipB in *Pichia pastoris* Glycerol, 30 °C	Lipase B (8.67 U mg^−1^)	[210]
*Candida sake*	Low glucose using *Tempranillo* must, 12 °C	Sorbitol (13 g L^−1^)	[211]
*Cystofilobasidium capitatum*	Glucose, starch	α-Amylases (0.76–1.73 U mL^−1^) Pectinases (0.5–1.57 U mL^−1^)	[132,212]
*Debaryomyces hansenii*	Fermented sausage L-arabinose, 28 °C Rapeseed straw supplemented with different carbon sources Brewery’s spent grain hydrolysate supplemented with yeast extract Rich medium with olive oil, Tween 80, pH 6.4	Prolyl aminopeptidase (0.034–416.25 mg) β-glucosidase (9 mU mL^−1^) Xylitol (0.42 g L^−1^ h) Ethanol (0.24 g L^−1^ h) Xylitol (0.36 g L^−1^ h) Lipases (7.44 U mL^−1^)	[213,214,215,216,217,218]
*Glaciozyma antarctica*	Short-chain soluble esters	Antifreeze proteins, lipases, esterases, glycosidases, proteases, chitinases, dienelactone hydrolases (1.15 U mg^−1^)	[196,219]
*Kluyveromyces lactis*	Glucose, lactose	α-Amylase (0.527 U mL^−1^) α-Galactosidase (2 mg L^−1^)	[220]
*Kluyveromyces marxianus*	Lactose or glucose High temperature Low oxygen levels	Bioethanol (10–90 mg mL^−1^)	[192,193]
*Leucosporidium scottii*	Glucose, saccharose, fructose Low temperature	Siderophores (1–2 mm)	[221]
*Mrakia blollopis*	Glucose, 22 °C Tween 80, yeast extract	Amylase (98–148 UA) Cellulases (151–165 UA) Lipase (51.7 U mg^−1^)	[222,223]
*Papiliotrema laurentii*	Glucose, nitrogen limitation	Oleic acid (5.9 g L^−1^)	[224]
*Rhodotorula glacialis*	Glucose, pH 5.4–6.2, 22 °C Glucose (12%), 10 °C	Amylases (132–220 UA) Oleic acid, linoleic, α-linoleic (22 g L^−1^)	[222,225]
*Rhodotorula glutinis*	Yeast Malt Broth, 30 °C	β-Carotene (57%) Torulene (33%) Torularhodin (10%) Total of carotenoids (0.266 mg g^−1^)	[197]
*Rhodotorula mucilaginosa*	Glucose, 28 °C pH 5–9	Inulinase for fructooligosaccharides production (250 g L^−1^) β-carotene (20.9 mg g^−1^) Oxalic acid (83.6–90.3 mg 100 mL^−1^) Gallic acid (0.5 mg g^−1^)	[226,227]
*Rhodotorula toruloides*	Different carbon sources Nitrogen limitation	Triacylglycerols, fatty acids (39 g L^−1^) β-Carotene, torulene, torularhodin (0.48–0.5 mg g^−1^) Terpenoids (bisabolene, 521–680 mg L^−1^)	[203,207,228]
*Tetracladium* sp.	Glucose, carboxymethylcellulose soluble starch	Cellulase (325 mm mg^−1^) Glucoamylase (1119 mm mg^−1^)	[222,229]
*Yarrowia lipolytica*	Glucose, xylose, agave bagasse hydrolysate	Fatty alcohols (205.4 mg L^−1^) Alkanes, Alkenes (23.3 mg L^−1^) Triacylglycerols, sterol esters, phospholipids (15 g L^−1^)	[26,230]
*Zygosaccharomyces bailii*	Glucose, fructose Sorghum extract High temperature	Farnesol, geraniol, nonanol nerolidol-2 (0.016 mg L^−1^) Esters (0.22 mg L^−1^) Organic acids (0.48 mg L^−1^) Aldehydes (0.87 mg L^−1^)	[190,191]
*Zygosaccharomyces rouxii*	Glucose, mannose, xylitol	Rose honey aroma (1.79–3.58 g L^−1^) Ethanol, ethyl propanoate, 1-butanol, ethyl 2-methylpropanoate 4-hydroxy-2-ethyl-5-methyl-3(2H)-furanone (1.7–2.1 mg L^−1^)	[189,231]

## Supplementary Materials

The following supporting information can be downloaded at: https://www.mdpi.com/article/10.3390/microorganisms10040794/s1, Table S1: Yeasts isolated from extreme environmental conditions. Table S2: Isolation of yeasts that inhabit extreme environments using different media and parameters. References [232,233,234,235,236,237,238,239,240,241,242,243,244,245,246,247,248,249,250,251,252,253,254,255,256,257,258,259,260,261,262,263,264,265,266,267,268,269,270,271,272,273,274,275,276,277,278,279,280,281,282,283,284,285,286] are cited in the supplementary materials.
