# Supplementary material for: Yeasts Inhabiting Extreme Environments and Their Biotechnological Applications
Source: Microorganisms. 2022 Apr 9;10(4):794. doi: 10.3390/microorganisms10040794 (PMC9028089; doi:10.3390/microorganisms10040794)
Supplement: Supplementary file 1 [file microorganisms-10-00794-s001.zip › microorganisms-1615132-supplementary.pdf]

Table S1. Yeasts isolated from extreme environmental conditions.

Genus or species	References
**Ascomycetes (cold condition)**<br>*Aureobasidium, Babjeviella sp., Candida sp., Candida parapsilosis, C. psychrophila, C. saitoana, C. sake, Debaryomyces, Dipodascus australiensis, Diutina rugosa, Exophiala xenobiotica, Galactomyces, Lipomyces, Metschnikowia australis, M. bicuspidata, Meyerozyma, Nadsonia, Pichia, Schizoblastosporion, Taphrina antarctica, Wickerhamomyces anomalus*<br>**Basidiomycetes (cold condition)**<br>*Apiotrichum sp., Bannozyma arctica, Bulleromyces sp., Cryptococcus sp., Curvibasidium sp., C. minutum, C. pallidum, Cutaneotrichosporon sp., C. cutaneum, C. moniliiforme, Cystofilobasidium sp., C. infirmominiatum, C. macerans, Dioszegia sp., D. antarctica, D. cryoxerica, D. fristingensis, D. hungarica, Filobasidium sp., F. oeirense, F. uniguttulatum, Glaciozyma watsonii, Goffeauzyma sp., G. gastrica, G. gilvescens, Holtermanniella sp., Itersonilia sp., I. pannonica, Krasilnikovozyma sp., Leucosporidium sp., L. creatinivorum, L. fragarium, Malassezia restricta, Mrakia sp., M. blollopis, M. frigida, M. gelida, Naganishia sp., N. adeliensis, N. albidosimilis, N. antarctica, N. liquefaciens, Phenoliferia sp., P. laurentii, P. glacialis, Rhodotorula sp., R. diobovata, R. glutinis, R. laryngis, R. mucilaginosa, Saitozyma sp., Solicoccozyma sp., S. terricola, S. metaroseus, S. salmonicolor, Sporobolomyces sp., S. johnsonii, Symmetrospora symmetrica, Tausonia sp., Vanrija sp., Vishniacozyma sp., V. carnescens, V. victoriae, Yamadamyces sp.*	[6,12,52,53,55,56,60,125,232–244]
**Ascomycetes (heat condition)**<br>*Candida blankii, C. freyschussii, Cyberlindnera mississippiensis, Exophiala capensis, Kazachstania bovina, K. telluris, Kluyveromyces marxianus, Lachancea thermotolerans, Ogataea angusta, O. thermophila, Sporopachydermia, Trichocladium griseum*<br>**Basidiomycetes (heat condition)**<br>*Cryptococcus neoformans, Cystobasidium, Holtermanniella wattica, Malassezia, Naganishia friedmannii, Rhodotorula toruloides, Takashimella tepidaria*	[8,12,41,51,52,58,59,240–246]
**Ascomycetes (dry condition)**<br>*Aureobasidium namibiae, Candida halonitratophila, C. thaimueangensis, Cladosporium sphaerospermum, Hanseniaspora opuntiae, H. uvarum, Lipomyces, Starmerella apicola, Wickerhamiella versatilis, Zygosaccharomyces bisporus*<br>**Basidiomycetes (dry condition)**<br>*Cryptococcus, Holtermanniella wattica, Naganishia albida, N. friedmannii, N. vishniacii, Rhodotorula toruloides, R. mucilaginosa, Solicoccozyma, Sporobolomyces johnsonii, S. roseus, Wallemia ichthyophaga, W. muriae*	[6,12,41,56,66,69,247]
**Ascomycetes (acidic condition)**<br>*Barnettozyma, Candida austromarina, C. dendrica, C. digboiensis, C. fluviatilis, C. oleophila, C. pseudoglaebosa, Cyberlindnera saturnus, Cyniclomyces guttulatus, Kazachstania exigua, Metschnikowia rancensis, Nadsonia starkeyi-henricii, Pichia membranifaciens, P. nakasei, Starmerella stellata, Wickerhamomyces anomalus, Wickerhamiella sorbophila, Zygosaccharomyces rouxii*<br>**Basidiomycetes (acidic condition)**<br>*Apiotrichum dulcitum, Buckleyzyma aurantiaca, Bulleromyces sp., Holtermannia, Leucosporidium muscorum, Naematelia, Papiliotrema, Pseudohyphozyma, Rhodotorula glutinis, R. toruloides, Saitozyma flava, Slooffia tsugae, Takashimella, Tremella fuciformis, Yunzhangia auriculariae*	[12,76,77,206,248–250]
**Ascomycetes (alkaline condition)**<br>*Aureobasidium pullulans, Barnettozyma, B. wickerhamii, Blastobotrys chiropterorum, Citeromyces matritensis, Clavispora lusitaniae, Cyberlindnera, Debaryomyces castellii, D. nepalensis, Diutina, Hyphopichia homilentoma, Komagataella pastoris, Kuraishia capsulata, Lachancea kluyveri, Lodderomyces elongisporus, Metschnikowia pulcherrima, Nadsonia, Nakazawaea holstii, Pachysolen tannophilus, Trichomonascus ciferrii, Wickerhamia fluorescens, Wickerhamomyces anomalus, Yamadazyma mexicana*<br>**Basidiomycetes (alkaline condition)**<br>*Cutaneotrichosporon cutaneum, Filobasidium, Hannaella luteola, Naganishia albida, Papiliotrema laurentii, Phaeotremella skinneri, Rhodosporidiobolus ruineniae, Rhodotorula glutinis, R. mucilaginosa, Sakaguchia dacryoidea, Sampaiozyma ingeniosa, Slooffia tsugae, Sporidiobolus pararoseus, Sporobolomyces roseus, Sterigmatomyces elviae*	[15,25,66,77]

**Ascomycetes (saline condition)**<br>*Aureobasidium, Candida, Citeromyces matritensis, Debaryomyces hansenii, D. subglobosus, Geotrichum candidum, Hortaea werneckii, Kluyveromyces, Metschnikowia, Millerozyma farinosa, Neophaeotheca triangularis, Pichia, Saccharomyces cereviaise, Schwanniomyces etchellsii, Trimmatostroma salinum, Williopsis, Yarrowia lipolytica*<br>**Basidiomycetes (saline condition)**<br>*Bandonia marina*, *Cryptococcus spp., Cutaneotrichosporon, Cystobasidium, Filobasidium, Leucosporidium, Malassezia restricta, Mrakia, Naganishia, Papiliotrema taeanensis, Phenoliferia, Pseudozyma, Rhodotorula, Saitozyma podzolica, Solicoccozyma terricola, Sporidiobolus, Sterigmatomyces halophilus, Trichosporon, Vanrija, Vishniacozyma carnescens, Wallemia ichthyophaga*	[12,13,17,18,66,86,215,234,252–261]
**Ascomycetes (osmotic condition)**<br>*Aureobasidium namibiae, Candida glucosophila, C. thaimueangensis, Debaryomyces hansenii, Hanseniaspora opuntiae, H. uvarum, Hortaea werneckii, Metschnikowia, Meyerozyma, Millerozyma farinosa, Pichia, Saccharomyces, Starmerella apicola, S. bacillaris, Wickerhamomyces, Zygosaccharomyces favi, Z. lentus, Z. mellis*<br>**Basidiomycetes (osmotic condition)**<br>*Sporobolomyces johnsonii, Wallemia ichthyophaga*	[69,71,72,75,79, 83,94,262]
**Ascomycetes (high incidence of UV)**<br>*Exophiala, Taphrina*<br>**Basidiomycetes (high incidence of UV)**<br>*Cryptococcus neoformans, Cystobasidium, Cystofilobasidium, Dioszegia, Naganishia friedmannii, Phaffia rhodozyma, Rhodotorula toruloides, R. mucilaginosa, Sporobolomyces*	[6,16,41,263–266]
**Ascomycetes (metal toxicity)**<br>*Aureobasidium pullulans, Candida, Epicoccum nigrum, Lachancea thermotolerans, Lodderomyces elongisporus, Yarrowia lipolytica, Zygosaccharomyces rouxii*<br>**Basidiomycetes (metal toxicity)**<br>*Cryptococcus, Papiliotrema, Rhodotorula mucilaginosa, Sporobolomyces roseus*	[12,99,267–271]
**Ascomycetes (cold, dry, UV)**<br>*Exophiala spp.*<br>**Basidiomycetes (cold, dry, UV)**<br>*Naganishia vishniacii, Rhodotorula mucilaginosa, R. frigidialcoholis* (*R. JG1b*)	[16,21,41,57,125,247]
**Ascomycetes (saline, osmotic, dry)**<br>*Aureobasidium melanogenum*, *A. namibiae*, *A. pullulans*, *A. subglaciale*, *Cladosporium*, *Hortaea werneckii*<br>**Basidiomycetes (saline, osmotic, dry)**<br>*Wallemia spp.*, *W. ichthyophaga*, *W. muriae*,	[67,68,80,86,272]
**Ascomycetes (cold, UV, metal toxicity)**<br>*Aureobasidium pullulans*<br>**Basidiomycetes (cold, UV, metal toxicity)**<br>*Rhodotorula mucilaginosa*	[270,273–277]
**Ascomycetes (oligotrophy, osmotic, cold, dry, UV)**<br>*Hortaea werneckii, Exophiala sp., Taphrina antarctica*<br>**Basidiomycetes (oligotrophy, osmotic, cold, dry, UV)**<br>*Naganishia antarctica, N. friedmannii, Rhodotorula mucilaginosa*	[19,109,278,279]
**Ascomycetes (oligotrophy, osmotic, different temperatures, UV)**<br>*Cryomyces antarcticus, C. minteri, Elasticomyces elasticus, Exophiala mesophila, Extremus antarcticus, Friedmanniomyces endolithicus, F. simplex, Meristemomyces frigidus, Oleoguttula mirabilis, Rachicladosporium sp., R. antarcticum, R. mcmurdoi, Recurvomyces mirabilis, Salinomyces thailandica, Taphrina antarctica, Vermiconia antarctica*<br>**Basidiomycetes (oligotrophy, osmotic, different temperatures, UV)**<br>*Naganishia friedmannii, N. vishniacii, Rhodotorula*	[19,109,278,280–284]

Table S2. Isolation of yeasts that inhabit extreme environments using different media and parameters.

Conditions	Culture media	Incubation	Extreme Yeasts	References
Cold	Yeast Peptone Dextrose	4-15 °C (up to 8 weeks)	*Cryptococcus, Dioszegia, Leucosporidium*	
	Malt Yeast Extract	5-20 °C (up to 4-8 weeks)	*Cryptococcus, Exophiala, Rhodotorula*	[233,236,282]
			Hortaea werneckii	
Heat	Sabouraud agar	25-30 °C (8 weeks) or up to 30 °C	*Takashimella tepidaria*	[59,279]
	Malt Yeast Extract		*Cryptococcus*	
Acidic	Malt Yeast Extract Peptone	18 °C, pH 3-5.7 (weeks)	*Candida, Cryptococcus, Rhodotorula*	[206,250]
	Peptone Yeast Extract Glucose	32 ± 2 °C, pH 2-6 (weeks)	*Candida*	
Alkaline	Peptone Yeast Extract Glucose K_2HP0_4 (50 mM) and NaCl (100 mM)	pH 5.7 (3 days), then pH 7-8, 8.5, 9-10.5 (weeks)	*Candida, Cryptococcus, Debaryomyces, Nadsonia, Rhodotorula*	[251,285]
	Peptone Yeast Extract Glucose (up to 33% NaCl)	> pH 9 (weeks)	*Candida*	
Saline	Seawater nutrient agar medium NaCl (2%), KCl (0.035%)	20 °C, pH 4.5 (5 days)	*Aureobasidium pullulans, Candida*	
	Malt Yeast Extract dissolved in artificial seawater NaCl (3%)	5–10 °C (2 weeks), then 20 °C (4 weeks)	*Kluyveromyces nonfermentans Candida tropicalis, Pichia*	
	Isolation medium with seawater	25 °C, pH 5.6 (10 days)	*Yarrowia lipolytica*	
	Yeast Peptone Dextrose plus NaCl (4%)	25-30 °C (weeks)	*Hortaea werneckii*	[13,80,86,209,258,286]
	Corn Meal Agar with seawater			
	Yeast Extract with saline water	25 °C (1-4 weeks)	*Aureobasidium pullulans*	
			Hortaea werneckii	
	Malt Extract Agar plus NaCl (17, 24, 32%)			
Osmotic	Yeast Nitrogen Base plus 2-(N-Morpholino) ethane sulfonic acid (0.1 M), containing NaCl (0.5-2 M)	28 °C, pH 6.0 (weeks)	*Debaryomyces hansenii*	
	Malt Extract Medium adjusted with various concentrations of plus NaCl	22 °C (2 weeks)	*Hortaea werneckii*	[79,83,262]

	Glucose Peptone Yeast Extract: (50% glucose)	25 °C (1 week)	*Zygosaccharomyces favi*	
Metal Toxicity	Yeast Peptone Dextrose supplemented with various concentrations of different heavy metal salts: $K_2Cr_2O_7$, $CrCl_3$, $Pb(NO_3)_2$, $ZnSO_4$, $CuCl_2$ $2H_2O$, $NiCl_2$ $6H_2O$, $CdCl_2$, and $Na_2HAsO_4.7H_2O$ (0.1-100 mM)	25-28 °C (1 week)	*Yarrowia lipolytica*	
	Minimal Salt Medium with salts of heavy metals ions ($CdCl_2$, $NaAsO_2$, $CuSO_4$, Pb $(NO_3)_2$ and $K_2Cr_2O_7$) (0.1 mM concentration each separately)	30 °C, pH 7 (2-3 days)	*Candida*	[99,269,270]
	Lysogeny Broth with Hg^{2+} (20 mg L^{-1}) in the form of $HgCl_2$ <br>Yeast Peptone Dextrose supplemented with Hg^{2+} (20–100 mg L^{-1})	30 °C, pH 7.5 (1 week)	*Rhodotorula mucilaginosa*	
